# The enemy within: lipid asymmetry in intracellular parasite–host interactions

**DOI:** 10.1042/ETLS20220089

**Published:** 2023-02-23

**Authors:** Merryn Fraser, Kai Matuschewski, Alexander G. Maier

**Affiliations:** 1 Research School of Biology, Australian National University, Canberra, Australia; 2 Department of Molecular Parasitology, Institute of Biology, Humboldt University, Berlin, Germany

**Keywords:** host–parasite interactions, lipid asymmetry, lipid metabolism, malaria, membranes

## Abstract

Eukaryotic pathogens with an intracellular parasitic lifestyle are shielded from extracellular threats during replication and growth. In addition to many nutrients, parasites scavenge host cell lipids to establish complex membrane structures inside their host cells. To counteract the disturbance of the host cell plasma membrane they have evolved strategies to regulate phospholipid asymmetry. In this review, the function and importance of lipid asymmetry in the interactions of intracellular protozoan parasites with the target and immune cells of the host are highlighted. The malaria parasite *Plasmodium* infects red blood cells and extensively refurbishes these terminally differentiated cells. Cholesterol depletion and an altered intracellular calcium ion homeostasis can lead to disruption in erythrocyte membrane asymmetry and increased exposure of phosphatidylserine (PS). Binding to the PS receptor on monocytes and macrophages results in phagocytosis and destruction of infected erythrocytes. *Leishmania* parasites display apoptotic mimicry by actively enhancing PS exposure on their surface to trigger increased infection of macrophages. In extracellular *Toxoplasma gondii* a P4-type ATPase/CDC50 co-chaperone pair functions as a flippase important for exocytosis of specialised secretory organelles. Identification and functional analysis of parasite lipid-translocating proteins, i.e. flippases, floppases, and scramblases, will be central for the recognition of the molecular mechanisms of parasite/host interactions. Ultimately, a better understanding of parasitic diseases, host immunity, and immune escape by parasites require more research on the dynamics of phospholipid bilayers of parasites and the infected host cell.

## Introduction

Many important pathogens, such as *Plasmodium spp.*, the causative agent of malaria, or *Leishmania spp.*, which cause a spectrum of skin, muco-cutaneous, and visceral diseases, are single-cell eukaryotes, which adopted a parasitic lifestyle. These protozoan parasites share all hallmarks of eukarya, including large genomes, cellular organelles, and complex phospholipid bilayers in their plasma membranes (PMs). Beyond barrier functions and nutrient uptake, the parasite PM facilitates pathogen/host interactions through modulation of immune cells and, for intracellular parasites, by driving host cell invasion. Accordingly, analysing the dynamics of parasite and parasitised phospholipid bilayers is central for a better understanding of disease, host immunity, and immune escape by the parasite.

**Figure 1.**
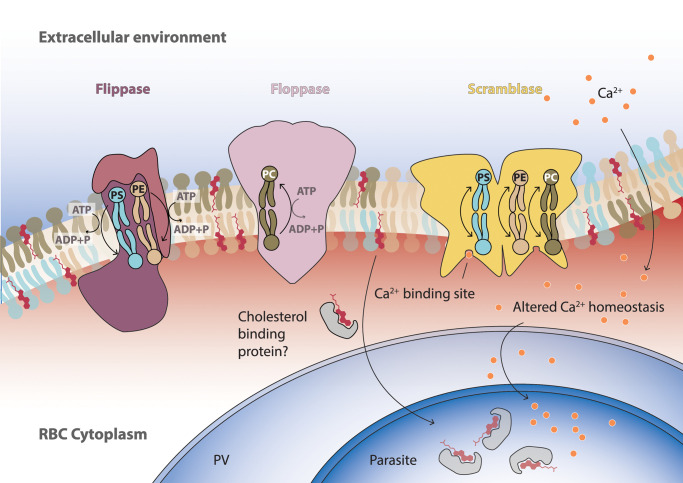
Dynamics of phospholipid asymmetry and cholesterol uptake upon parasite infection exemplified by a lipid bilayer of a *Plasmodium*-infected erythrocyte. Shown are major phospholipid species, the sterol cholesterol, Ca^2+^, and candidate membrane-bound enzymes that catalyse phospholipid shuttling between the outer and inner lipid leaflet. Enrichment of phosphatidylserine (PS; blue) and phosphatidylethanolamine (PE; cream) in the interior lipid sheet is catalysed by a flippase (purple) under ATP hydrolysis. In contrast, the exterior layer facing the blood stream, endothelial cells and phagocytes is enriched in phosphatidylcholine (PC; brown) by an ATP-hydrolysing floppase (pink). Cholesterol (red) is sequestered by the growing parasite, whereas Ca^2+^ homeostasis is modulated in infected red blood cells. Activation of scramblase (yellow) by an increase in intracellular Ca^2+^ results in the random shuttling of all three phospholipids across the two layers. Enrichment of host-derived cholesterol might be mediated by a cholesterol-binding protein. ADP, adenosine diphosphate; ATP, adenosine triphosphate. Shape of proteins is based on structures of representative flippase (P4-ATPase Drs2p-CDC50p [1]), floppase (ABCG2 [2]), and scramblase molecules (TMEM16F [3]) deposited in the protein data bank (PDB [4]).

Red blood cells (RBCs) maintain an asymmetry between the lipids present in each layer of their membrane bilayer (Figure 1). Anionic phospholipids such as phosphatidylserine (PS) and phosphatidylethanolamine (PE) are sequestered to the inner (cytoplasmic) leaflet of the membrane, while other lipids, such as phosphatidylcholine (PC) are more abundant in the outer (extracellular) leaflet [5]. Indeed, the concept of membrane asymmetry was first described in RBCs nearly half a century ago [6,7]. Technological advancements and concentrated research efforts have vastly improved our knowledge and understanding of membrane asymmetry since these early studies. We now know that membrane asymmetry is not restricted to RBCs, but a feature of all mammalian cells and across the different kingdoms of life, and of organelle membranes, too [8–13]. Membrane asymmetry is important for a whole range of cellular functions — from membrane biophysical properties, protein–membrane interactions, and cell-to-cell interactions, including as a principal signal to initiate apoptosis acting as an ‘eat me’ label to a neighbouring cell, which will initiate phagocytic uptake [14,15]. This review will concentrate on the function and importance of lipid asymmetry in the interactions of intracellular protozoan parasites with the target and immune cells of the host, with a particular emphasis on the malaria parasite *Plasmodium*, which infects RBCs.

## Lipid asymmetry regulation in red blood cells

Phospholipid asymmetry is established, regulated, and ultimately collapsed by three classes of lipid-translocating enzymes, termed flippases, floppases, and scramblases (Figure 1) [5,16,17]. These terms are used as broad descriptors of multiple proteins that perform the respective functions. Flippases move anionic phospholipids, particularly PS and with lower affinity PE, from the outer layer to the inner layer in an ATP-dependent manner [5,18]. This unidirectional movement of these negatively charged lipids creates and maintains the unequal distribution between the bilayers (Figure 1). The most studied flippases consist of members of the P4-type ATPase protein family and an accessory CDC50 subunit, which is located at the outer layer and likely regulates the binding of the lipid substrate by acting both as a chaperone and integral part of the lipid-translocating machinery [18]. P4-ATPases localise to the PM and intracellular membranes and display different substrate selectivity for the aminophospholipids PS, PE, or PC, but also a broader range of lipid substrates, such as lysophospholipids. Thus, identifying bona fide flippases among the range of P4-ATPase members necessitates biochemical assays and experimental genetics. In human RBCs ATP11C is the most abundant P4-type ATPase, followed by ATP11A and ATP11B [19–21]. Forward and reverse genetic approaches in humans and mice, respectively, have revealed the functional importance of ATP11C for maintaining membrane asymmetry in RBCs and other cell types, and its mutation can lead to altered cell development and lifespan [22–24]. Floppases similarly function in an ATP-dependent manner, but instead shuttle lipids such as PC to the outer layer [5] (Figure 1). Several ATP-binding cassette (ABC) proteins have been ascribed floppase function, such as ABCC1, ABCA7, and ABCG2, which are present in RBCs [19,21,25,26].

The third class of these enzymes, scramblases, are responsible for the disruption of lipid asymmetry [5] (Figure 1). They do not require ATP to function, but instead indiscriminately scramble lipids in both directions, allowing the membrane to reach a symmetric equilibrium [5]. Scramblase activity is generally repressed in cell membranes to ensure that asymmetry is maintained, and only activated under specific circumstances — by increased intracellular calcium, cholesterol depletion, or by caspase-mediated cleavage; these circumstances can occur in response to cell damage, signalling cascades, or during apoptosis [5,21,27–29]. PS exposure can act as an ‘eat me’ signal to phagocytes, such as monocytes and macrophages, which express PS receptors on their surfaces, in order to engulf and clear away these damaged or apoptotic cells [30,31]. Scramblase activation and subsequent PS exposure are also a crucial part of platelet activation [5,27]. Several proteins and their family members are suggested to act as scramblases, such as TMEM16F (involved in platelet activation), XKr8 (involved in caspase-mediated apoptotic PS exposure), and PLSCR1–4 [21,32–34]. The annotation of the latter has received scrutiny over whether it truly acts as a scramblase under physiological conditions, but there are still suggestions that it could play a role [21,28,35]. In RBCs, PLSCR1 and PLSCR4 are highly abundant, TMEM16F levels are low, and XKr8 appears to be absent [19,21].

The regulation of programmed cell death (or apoptosis) is an important mechanism for multicellular organisms to remove unwanted cells during development or damaged cells during infection or senescence, with phagocytic cells such as macrophages and monocytes participating in this process [31,36]. Since RBCs do not contain a nucleus, mitochondria, or protein synthesis machinery, they constitute elementary cells and have been a popular model system for membrane research. However, this also means that RBCs are not able to perform the same apoptotic processes as nucleated cells, such as the fragmentation of DNA and mitochondrial membranes [36,37]. Nonetheless, RBCs can enter an apoptotic-like state, termed ‘eryptosis,’ which shares other common markers of apoptosis — like altered ion balance, particularly of calcium and potassium, cell shrinkage, membrane blebbing, and a loss of membrane asymmetry, resulting in PS exposure [38,39]. Indeed, RBCs may be particularly dependent on PS exposure as a signal for cell death, since they lack the other apoptotic signals available to nucleated cells. These eryptotic markers generally begin to appear as the RBC ages, when factors such as ATP depletion hamper the cells’ ability to actively maintain ion homeostasis, water balance, and deformability [40,41]. These changes signal that the aging RBC is ready to be removed from circulation, and it is generally filtered out and phagocytosed in the spleen or liver [42].

## The case of intracellular parasites: refurbishing host cells

Several parasites have chosen RBCs as their home, since they provide abundant nutrients, shelter from the host's immune system, and transportation through the human body. These parasites are members of the phylum apicomplexa, which are all obligate intracellular pathogens and infect a wide variety of host cells [43]. This phylum encompasses parasites such as *Plasmodium*, the causative agent of malaria, *Babesia* and *Theileria*, tick-borne pathogens of veterinary importance [44–46]. As the parasite grows inside, it modifies the RBC to support its survival — scavenging lipids, proteins, and other molecules from the host cell and extracellular environment. Parasites introduce new channels and virulence factors in the RBC membrane to ensure adequate nutrient uptake and aid in the avoidance of the immune system [47,48]. Since the parasite seemingly places a heavy burden on the RBC — even before it completes its replication and ultimately destroys the cell to seek out new host cells — many researchers have sought to investigate whether these consequences include changes to membrane asymmetry.

## Asymmetry at the *Plasmodium*-infected RBC membrane: cell culture artefacts and clinical relevance

Many studies have attempted to determine whether lipid asymmetry in the RBC membrane is disrupted when parasitised with *Plasmodium*, often reaching contradictory results (for example, [49–54]). A recent meta-analysis of these studies looked at the contradictory body of evidence for whether or not PS is exposed on the surface of infected RBCs [22]. A main conclusion was that much of the controversy is likely attributable to the variety of methods to assay PS exposure — lipid hydrolysis, anti-PS antibodies, thrombin-activation, and eventually annexin V staining — and the shortcomings associated with each [22]. In addition, several of the experimental conditions might have artificially exacerbated PS exposure — glucose deprivation, high potassium concentrations, extreme parasite burdens — suggesting that observed disruptions to lipid asymmetry may not necessarily reflect physiological conditions [22]. More recent techniques, such as combined annexin V and DNA staining to distinguish infected and uninfected RBCs without artificial separation, have addressed many of these arguments [22].

On the balance of evidence, PS is likely exposed in a portion of infected RBCs under *in vitro* culture conditions, and this disruption to membrane asymmetry can be exacerbated by a number of factors such as heat stress (mimicking malarial fevers), hyperparasitaemia (leading to an increase in parasite burden), and treatment with chemical agents, which directly or indirectly lead to infected RBCs entering eryptosis [52,54–56].

Two mechanisms have been identified as the source for this disruption in membrane asymmetry (Figure 1): an increase in calcium levels in the otherwise low-calcium environment of the RBC cytoplasm, and cholesterol depletion from the RBC membrane — both of which can activate scramblase proteins and lead to PS exposure [28,29,57]. The infected RBC population has a higher level of scramblase activity compared to uninfected RBCs, although a concurrent increase in flippase activity has also been observed [54]. It has been hypothesised that cholesterol depletion likely plays a role in both phenomena, since this lipid affects membrane fluidity and can, therefore, affect the turnover rate of these proteins [28,29,58,59].

## Asymmetry of internal membranes: more questions than answers

**Figure 2.**
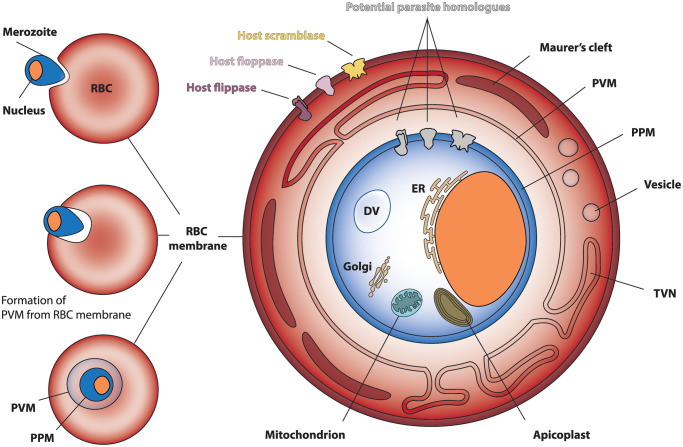
Establishment of multiple membrane structures in a *Plasmodium falciparum*-infected erythrocyte. Shown are the invagination of the host cell plasma membrane during parasite invasion resulting in a parasite-derived organelle, the parasitophorous vacuole, and the intracellular growth stage of *P. falciparum* in an infected erythrocyte. Host-encoded flippases (purple), floppases (pink), and scramblase (yellow) are highlighted and potential parasite homologues are shown in grey. Note multiple parasite organelles, which include the classic eukaryotic single membrane organelles, ER and Golgi cistern, the double membrane organelles, nucleus (orange) and mitochondrion, a specialised lysosome (termed digestive vacuole), and a relict plastid organelle. The latter, termed the apicoplast, is non-photosynthetic, and contains four lipid membranes, which originate from the secondary endosymbiosis of a protist algae. In addition, the growing parasite induces membranous structures in the host cell, including vesicles, a tubovesicular network, and Maurer's clefts. DV, digestive vacuole; ER, endoplasmic reticulum; PPM, parasite plasma membrane; PVM, parasitophorous vacuole membrane; RBC, red blood cell/erythrocyte; TVN, tubovesicular network.

While mature human RBCs lack internal membranous structures, parasitic invasion creates a complex structure of membranes, where asymmetry could play a role in parasite survival (Figure 2). *Plasmodium* parasites invade RBCs using a variety of receptors and ligands, with signalling cascades in both parasite and host cell culminating in the invagination of the RBC membrane around the parasite [60,61]. The parasite remains in this resulting membrane-bound organelle, termed the parasitophorous vacuole (PV), adding newly synthesised lipids and proteins as it grows and multiplies, until the daughter cells (merozoites) are ready to egress both the PV and the host cell before invading new RBCs [47,62–64]. As a consequence, molecules that are exchanged between the host serum and the parasite have to transverse three membranes: RBC plasma membrane, PV membrane (PVM), and parasite plasma membrane (PPM).

One notable phenomenon is that the PVM membrane is created from the ‘outside in’ host cell membrane, and, accordingly, the proteins may also be orientated in the opposite direction [65,66]. This has been proposed as a mechanism for retaining high calcium concentrations in the vacuolar compartment, since the human calcium extrusion pump, PMCA, may now be orientated to pump calcium in this direction [65,66]. Similarly, erythrocyte flippases and floppases may be orientated and functioning in the new direction, keeping PS and PE on what is still technically the cytoplasmic side. However, not all host proteins are translocated into the PVM [67], and so the localisation, orientation, and function of lipid-translocating proteins in this membrane are yet to be confirmed.

In addition to the PVM, the parasite also establishes membranous structures within the host cell cytoplasm, such as the Maurer's clefts and tubovesicular network (TVN), which are involved in the trafficking of lipids and proteins between the host and parasite [68] (Figure 2). The parasite contains membrane-bound organelles originally derived from evolutionary endocytosis — the double-membraned mitochondria, and the quadruple-layered apicoplast [69,70]. This non-photosynthetic plastid likely resulted from the secondary endocytosis of algae, and compartmentalises processes, such as isoprenoid biosynthesis via methyl erythritol phosphate (MEP), fatty acid chain elongation, and parts of the haem biosynthetic pathway [71]. This multitude of membranes raises many potential questions about the existence, role, and maintenance of membrane asymmetry between these different compartments.

The overall lipid composition of the RBCs changes significantly upon infection with *Plasmodium*. As the parasite develops, it synthesises and scavenges lipids, increasing the amounts in total lipids, phospholipids, neutral lipids, sphingolipids, and cholesterol [71–74]. Changes to individual structures are much harder to measure, as protocols for separating the parasite from its host cell generally leave segments of the latter attached [75]. It is well established that the parasite PM normally contains a relatively low abundance of cholesterol, since it shows resistance to saponin lysis, while both the host PM and PVM can be lysed (though not fully removed) [75,76]. It has also been demonstrated that the PVM initially matches the cholesterol composition of the host cell membrane it was derived from, but this cholesterol is diluted as the PVM grows with the incorporation of parasite-synthesised phospholipids [64]. A lipidomic study of isolated apicoplasts also reveals an atypical lipid composition compared to the overall composition found in infected RBCs [77]. Apicoplasts are particularly rich in saturated phospholipids (in particular phosphatidylinositol) and contain also lipids that are rather unusual for plastids (like sphingomyelins, ceramides, and cholesterol). Collectively, these studies highlight clear differences between host and parasite membrane composition, however, none have probed the asymmetric state of any of these membranes. While the need to sequester certain lipids on either side of these internally hidden membranes may not be as important for intracellular parasites in the absence of phagocyte recognition, membrane asymmetry may have other functional roles at these sites, such as protein binding. Whether there are functional roles of asymmetry or differences between different compartments and organelles remains to be investigated.

In the absence of experimental evidence, we can only speculate about the asymmetrical nature of these membranes based on the existence of putative lipid-translocating proteins. The parasite genome encodes several proteins that show homology to human flippases, floppases, and scramblases. In the case of flippases, this includes P4-ATPases PF3D7_1219600 (*Pf*ATP2) and PF3D7_1223400 (*Pf*ATP8). These are likely essential to the parasite's survival, and orthologs in another *Plasmodium* species localise to the PPM [78–80]. *Plasmodiun falciparum* encodes three CDC50 orthologues, PF3D7_0719500 (*Pf*CDC50A), PF3D7_1133300 (*Pf*CDC50B), and PF3D7_1029400 (*Pf*CDC50C), the latter being the chaperone partner of *Pf*ATP2 [81,82]. Other P4-ATPases, such as PF3D7_031900 (*Pf*ATP7), may localise to internal membranes [80]. The parasite encodes 11 ABC family members containing transmembrane domains, of which PF3D7_0112200 (*Pf*MRP1/ABCC1), PF3D7_1229100 (*Pf*MRP2/ABCC2), PF3D7_1447900 (*Pf*MDR2/ABCB2), PF3D7_1339900 (*Pf*MDR5/ABCB5) have been localised to the PPM [81,83,84]. These proteins might operate as floppases, though no information is currently available to assign functions to any of these candidate transport proteins. A parasite candidate scramblase protein, PF3D7_1022700 (*Pf*PLSCR), was identified based on homology to the human PLSCR proteins, and displayed scramblase-like activity when expressed in proteoliposomes [85]. It appears to localise to membranes within the parasite during the majority of intraerythrocytic asexual development, with the potential exception of merozoites.

The existence of these proteins could indicate that parasite membranes are asymmetrically arranged, though further evidence is clearly required to determine the localisation and function of these proteins, Of note, none of the parasite-encoded, candidate lipid-translocating proteins has yet been localised to the PVM. None of the proteins contains common export motifs indicative of export beyond the PPM, but this does not preclude the possibility that they could be exported in a different manner [86]. There is a need to ultimately determine the distribution of lipids within different parasite compartments through studies, which probe the asymmetrical nature of internal membrane structures, preferably without destructive separation techniques. Towards this goal, organelle enrichment by affinity purification of epitope-tagged signature membrane proteins, as established for the apicoplast [77], is expected to reveal the organellar lipid composition and permit conventional cell biological assays, e.g. annexin V stain. Symmetric membranes are less common than asymmetric membranes, likely because they are less selective for transiting molecules. The superior selectivity together with the ‘scaffolding effect’, where different lipid compositions on both bilayers can provide greater mechanical stability, hold the membrane in its proper shape, and prevent it from deformation, makes it likely that the dynamic intracellular environments are reflected by asymmetric internal membranes.

## Consequences of altered asymmetry: phagocytosis of infected erythrocytes

**Figure 3.**
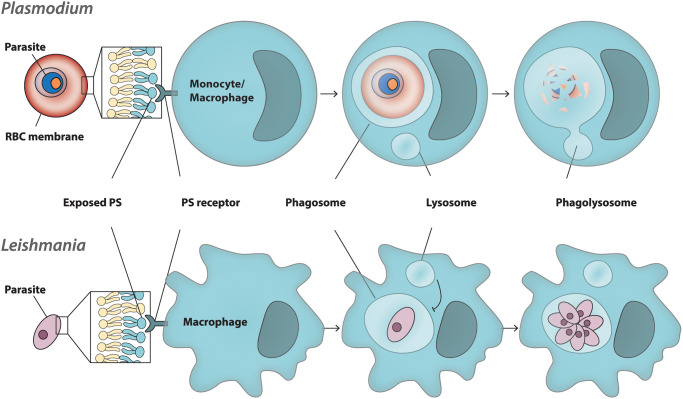
Alternative interference with phagocytosis by protozoan parasites. *Plasmodium*-infected cells (top) are recognised through the exposure of phosphatidylserine (PS) on the erythrocyte plasma membrane (and additional parasite-encoded proteins; not shown). Binding to the PS-receptor on macrophages results in phagocytosis and lysosomal fusion with the endocytic compartment leading to the formation of a phagosome. Killing inside a phagosome occurs through classical activation (M1) and is accompanied by a pro-inflammatory response. *Leishmania* parasites (bottom) enhance PS exposure on their surface to increase phagocytic uptake by macrophages, which constitute their only host cell for growth and replication. *Leishmania* parasites have evolved mechanisms to inhibit lysosomal fusion and/or acidification of the phagosome, resulting in a replication-competent niche inside infected macrophages.

Disruption to membrane asymmetry has several conceivable biological consequences, some of which have been explored experimentally. The essential nature of parasite proteins, such as *Pf*ATP2, suggests an important functional role during asexual growth. Due to lack of information about asymmetry in internal membranes, we will focus on the host cell membrane, where there is potential for interaction with immune cells, such as monocytes and macrophages, which recognise exposed PS as a signal for phagocytosis (Figure 3). There is *in vitro* evidence for a role of PS exposure in monocyte phagocytosis of infected RBCs, and these interactions can be blocked with either the PS-binding protein annexin V on iRBCs, or with PS liposomes on the phagocytes [54,87]. Similarly, blocking a phagocyte PS-receptor protein, CD36, decreases monocyte and macrophage phagocytosis of infected RBCs. However, since CD36 is a promiscuous ‘scavenger’ receptor, and also recognises other ligands on parasitised RBCs, this provides less direct evidence than inhibition of PS exposure on infected RBCs. In patient samples, monocytes with phagocytosed parasites are frequently observed (Figure 3). More broadly, monocytes and macrophages influence the course of malaria infection through their pro-inflammatory response after classical (M1) activation, which includes the release of IL-1, IL-12, IL-23, and chemokines [88,89]. Aside from phagocytosis, *in vitro* evidence has also suggested a role for PS exposure in parasite cytoadherence to ligands present on the vascular endothelium — an important part of the parasites’ defence against the host immune system, which sequesters them away from circulation and prevents splenic clearance [52,54,90]. While the importance of the avoidance strategy is well-accepted, a causal relationship between PS exposure and cytoadherence *in vivo* has not been established.

As outlined above, there are many gaps in our understanding of membrane asymmetry and its role in malaria infection. It is also clear that there is no simple answer for who benefits most from this process — a typical phenomenon of these close relationships between parasite and host, which have evolved together in a constant arms race [91]. Being able to recognise and phagocytose an infected cell may seem to be a simple benefit to the host, but if this comes at the cost of a hyperinflammatory response, then it is no longer advantageous [92,93]. Similarly, it may seem like immune evasion is the ultimate goal of a parasite, but perhaps the clearance of a subsection of parasites could be an advantage, since it lowers the chance of the host dying before the infection can be transmitted — an especially important consideration for *P. falciparum*, which requires 10–14 days to fully develop transmissible parasites [94]. It may, therefore, be beneficial for the parasites to reduce parasite burden, and recognition by exposed PS may trigger a less severe immune response than opsonisation by antibodies or complement [40,95]. Collapsing membrane asymmetry may also have other complex consequences in processes such as invasion, egress, and nutrient uptake. The threat of immune discovery could mean that the parasite is limited in which modifications it can induce in the RBC before collapsing asymmetry and hence impose some restrictions on the parasite. With this complicated relationship in mind, we will turn our attention to another intracellular parasite which appears to take advantage of PS exposure.

## Apoptotic mimicry and the *Leishmania* parasite

Like the apicomplexans, *Leishmania* is also a protozoan parasite, belonging to the class Kinetoplastida. This parasite is the causative agent of leishmaniasis, which affects the epithelia of skin, mucosa and inner organs. During chronic infections, *Leishmania* parasites reside in macrophages [96–99]. Rather than avoiding PS exposure and the detection by the immune system, these parasites exploit this mechanism to end up in their host cells (Figure 3). To aid in its uptake, the parasite uses ‘apoptotic mimicry’ — where it exposes PS in the outer layer of its PM and hence increasing phagocyte recognition [98–102]. After subsequent engulfment, the parasites employ a number of strategies to prevent their degradation, allowing them to live and multiply in the macrophage without being targeted by the immune system [98,103]. The molecular basis for enhanced PS exposure implicated in *Leishmania*-macrophage interaction is under investigation, but one candidate protein, LABCG2, a *Leishmania* member of ABC half-transporters (ABCG subfamily), has been implicated in PS exposure and macrophage infectivity of *Leishmania major* metacyclic promastigotes, the parasite stage transmitted by infected sand-flies (Phlebotominae) [104]. We note that some studies have failed to detect PS in the PM of *Leishmania donovani* promastigotes by complementary lipid analysis techniques [105,106]. Parasites were still stained with annexin V [106], illustrating that annexin V, and perhaps also phagocytic PS receptors, may not be completely specific to PS, despite consistent detection of PS on early apoptotic cells by fluorescently labelled annexin V [107].

Drug-induced increase in intracellular Ca^2+^ levels of *Leishmania* promastigotes, the replicative stage in the sandfly vector, induced swift, bi-directional and non-specific trans-bilayer movement of phospholipids indicative of scramblase activity [108,109], but a candidate scramblase has not yet been identified in *Leishmania* genomes [110]. Accordingly, functional analysis of *Leishmania* lipid-translocating proteins and more cell biological assays are needed to clarify the molecular basis of this phenomenon. Apoptotic mimicry has also been described for other parasites which can infect macrophages, such as *Toxoplasma gondii*, a food-borne apicomplexan parasite that causes toxoplasmosis in immune-suppressed individuals and foetuses, and *Trypanosoma cruzi*, the causative agent of Chagas disease and transmitted by kissing bugs, where it seems to promote an anti-inflammatory response [111,112]. More research is warranted to resolve whether apoptotic mimicry of parasites is an active regulatory mechanism that contributes to host colonisation *in vivo* or whether it can be at least partially attributed to experimental conditions during cell culture.

## Asymmetry and *Toxoplasma gondii*: flippase contributes to microneme secretion

Membrane asymmetry might also be important for infections by *T. gondii*, a universal parasite capable of infecting nucleated cells of a wide range of homeothermic hosts [113]. While data on potential interference with the dynamics of lipid asymmetry of *Toxoplasma*-infected cells are lacking, the import of PS and PE, but not PC, into extracellular parasites (so-called tachyzoites) has been reported [114]. *T. gondii* encodes six candidate P4-type ATPases and four CDC50 co-chaperomes, of which one pair, ATP2B and CDC50.4, mediates flipping of PS, which in turn acts as a lipid mediator for the last step in microneme exocytosis, i.e. fusion of micronemes with the PM [115]. Flippase activity was measured at the single-cell level employing a lactadherin C2 domain — GFP fusion protein as genetically encoded molecular probe [15]. These findings will inform corresponding work in malaria research, and, indeed, a recent experimental genetics analysis of the three *Plasmodium* CDC50 members uncovered roles in the maturation of blood stages and efficient parasite egress for CDC50C and CDC50B, respectively [82]. Candidate floppase and scramblase genes have not yet been assigned in the *T. gondii* genome [116], perhaps indicating that intracellular replication in nucleated cells might require less regulation of lipid asymmetry of host cell PMs than inside erythrocytes, which are refractory to *T. gondii* infection.

## Conclusions

While many aspects of membrane asymmetry have been elucidated in eukaryotic cells, there are still many questions about what happens when one eukaryote parasitises another. The added complexity of having two organisms with fundamentally different objectives, along with the deeply nested structure of so many membranes within membranes, presents additional challenges for those attempting to tease apart the organisation of the bilayers. However, this system — although complicated — allows for the exploration of the functional role of membrane asymmetry and the process of its regulation. We have highlighted some intriguing gaps in our collective understanding of membrane organisation in the context of host–parasite interactions, focusing mainly on the malaria parasite *Plasmodium*. Without a doubt, further investigations into this fascinating topic will reveal more ways how membrane asymmetry is used in the tug-of-war between the parasite and the host.

## Summary

- Intracellular parasites establish complex membrane structures inside host cells.
- Protozoan parasites encode candidate lipid-translocating enzymes, but data on the dynamics of lipid asymmetry on the plasma and internal membranes are scarce.
- Erythrocyte infections by malarial parasites lead to changes in lipid asymmetry on the RBC membrane and phagocytosis.
- *Leishmania* parasites use disrupted phospholipid asymmetry on their surface to enhance infection of macrophages by phagocytic uptake.
- A *Toxoplasma gondii* flippase mediates the final step in membrane fusion during exocytosis.

## Open Access

Open access for this article was enabled by the participation of Australian National University in an all-inclusive *Read & Publish* agreement with Portland Press and the Biochemical Society under a transformative agreement with CAUL.

## Abbreviations

ABC: ATP-binding cassette
PC: phosphatidylcholine
PE: phosphatidylethanolamine
PM: plasma membranes
PPM: parasite plasma membrane
PS: phosphatidylserine
PV: parasitophorous vacuole
PVM: PV membrane
RBCs: red blood cells
